# Treatment Strategies for GLILD in Common Variable Immunodeficiency: A Systematic Review

**DOI:** 10.3389/fimmu.2021.606099

**Published:** 2021-04-15

**Authors:** Olivia A. C. Lamers, Bas M. Smits, Helen Louisa Leavis, Godelieve J. de Bree, Charlotte Cunningham-Rundles, Virgil A. S. H. Dalm, Hsi-en Ho, John R. Hurst, Hanna IJspeert, Sabine M. P. J. Prevaes, Alex Robinson, Astrid C. van Stigt, Suzanne Terheggen-Lagro, Annick A. J. M. van de Ven, Klaus Warnatz, Janneke H. H. M. van de Wijgert, Joris van Montfrans

**Affiliations:** ^1^ Department of Pediatric Immunology and Rheumatology, Wilhelmina Children’s Hospital, Utrecht, Netherlands; ^2^ Department of Immunology and Rheumatology, University Medical Center Utrecht, Utrecht, Netherlands; ^3^ Department of Internal Medicine, Amsterdam University Medical Center, Amsterdam, Netherlands; ^4^ Department of Medicine, Division of Clinical Immunology and Department of Pediatrics, Mount Sinai Hospital, New York, NY, United States; ^5^ Department of Internal Medicine, Division of Clinical Immunology and Department of Immunology, Erasmus University Medical Center Rotterdam, Rotterdam, Netherlands; ^6^ UCL Respiratory, University College London, London, United Kingdom; ^7^ Wilhelmina Children’s Hospital, Department of Pediatric Pulmonology, Utrecht, Netherlands; ^8^ Department of Pediatric Pulmonology, Amsterdam University Medical Center, Amsterdam, Netherlands; ^9^ Departments of Rheumatology and Clinical Immunology, Internal Medicine and Allergology, University Medical Center Groningen, Groningen, Netherlands; ^10^ Department of Immunology, Universitätsklinikum Freiburg, Freiburg, Germany; ^11^ Department of Rheumatology and Clinical Immunology, Division of Immunodeficiency, Medical Center - University of Freiburg, Faculty of Medicine, Freiburg, Germany; ^12^ Julius Center for Health Sciences and Primary Care, University Medical Center Utrecht, Utrecht University, Utrecht, Netherlands

**Keywords:** systematic review, immunodeficiency, common variable immunodeficiency, CVID, granulomatous lymphocytic interstitial lung disease, GLILD, treatment

## Abstract

**Introduction:**

Besides recurrent infections, a proportion of patients with Common Variable Immunodeficiency Disorders (CVID) may suffer from immune dysregulation such as granulomatous-lymphocytic interstitial lung disease (GLILD). The optimal treatment of this complication is currently unknown. Experienced-based expert opinions have been produced, but a systematic review of published treatment studies is lacking.

**Goals:**

To summarize and synthesize the published literature on the efficacy of treatments for GLILD in CVID.

**Methods:**

We performed a systematic review using the PRISMA guidelines. Papers describing treatment and outcomes in CVID patients with radiographic and/or histologic evidence of GLILD were included. Treatment regimens and outcomes of treatment were summarized.

**Results:**

6124 papers were identified and 42, reporting information about 233 patients in total, were included for review. These papers described case series or small, uncontrolled studies of monotherapy with glucocorticoids or other immunosuppressants, rituximab monotherapy or rituximab plus azathioprine, abatacept, or hematopoietic stem cell transplantation (HSCT). Treatment response rates varied widely. Cross-study comparisons were complicated because different treatment regimens, follow-up periods, and outcome measures were used. There was a trend towards more frequent GLILD relapses in patients treated with corticosteroid monotherapy when compared to rituximab-containing treatment regimens based on qualitative endpoints. HSCT is a promising alternative to pharmacological treatment of GLILD, because it has the potential to not only contain symptoms, but also to resolve the underlying pathology. However, mortality, especially among immunocompromised patients, is high.

**Conclusions:**

We could not draw definitive conclusions regarding optimal pharmacological treatment for GLILD in CVID from the current literature since quantitative, well-controlled evidence was lacking. While HSCT might be considered a treatment option for GLILD in CVID, the risks related to the procedure are high. Our findings highlight the need for further research with uniform, objective and quantifiable endpoints. This should include international registries with standardized data collection including regular pulmonary function tests (with carbon monoxide-diffusion), uniform high-resolution chest CT radiographic scoring, and uniform treatment regimens, to facilitate comparison of treatment outcomes and ultimately randomized clinical trials.

## Introduction

Common variable immunodeficiency disorders (CVID) are the most common symptomatic primary immunodeficiencies, with an estimated incidence between 1:10.000 and 1:50.000 (1). Patients typically suffer from recurrent respiratory tract infections, such as bronchitis, sinusitis, otitis media and pneumonia. Moreover, they are often affected by immune dysregulation, a term which encompasses auto-immune manifestations, auto-inflammatory disease and lymphoproliferation, and by malignancy (2). Infection risk in CVID can be minimized by means of antimicrobial prophylaxis and immunoglobulin replacement therapy (IgRT). In contrast, immune dysregulation is much more difficult to prevent and treat, and remains a major cause of morbidity and mortality (3–6).

Granulomatous lymphocytic interstitial lung disease (GLILD) is one of the complications of CVID and is considered the pulmonary manifestation of multi-system immune dysregulation. GLILD occurs in approximately 10-20% of patients with CVID and was reported to be responsible for a reduction in life expectancy of more than 50% after diagnosis in adult patients, from a median of 28.9 to 13.7 years (6, 7). GLILD may be asymptomatic, or may present with non-specific symptoms such as cough and dyspnea on exertion (4). Small or large nodules, consolidations and ground glass abnormalities in the lower regions of the lung on high-resolution CT-scan are highly suggestive of GLILD (8). The diagnosis can be confirmed by biopsy (via video-assisted thoracoscopic surgery, transbronchial or percutaneous intervention) and FDG-PET-CT may be used for the identification of active inflammatory lesions elsewhere (4, 9). The combination of routine chest CT-scans and pulmonary function tests, including specifically diffusing capacity of carbon monoxide, should be used to identify GLILD in CVID and monitor disease progression (9).

The etiology of GLILD is still poorly understood. Maglione and colleagues pointed out that patients with X-linked agammaglobulinemia (XLA) have severe antibody deficiency that is even more pronounced than CVID but only rarely develop GLILD (10). Patients with XLA lack mature B-cells, whereas patients with CVID have peripheral B-cells, although often with impaired function, suggesting that B-lymphocytes may play a causative role in GLILD development. Indeed, lymphocytic (but not the granulomatous) progression has been associated with an increased production of B-cell activating factor (BAFF), which in turn leads to activation of the anti-apoptotic factor Bcl-2, thereby promoting B-cell survival as well as an increase of IgM producing CD21 low B-cells (10). Unger et al. linked the expansion of CD21low B-cells with disproportionally high numbers of Th1 cells and increased interferon-γ production, probably reflecting the aberrant combined T-B interaction in the pathogenesis of interstitial lung disease in CVID (11). It has also been suggested that viral infections may trigger GLILD, as Wheat et al. identified a correlation between human herpesvirus 8 (HHV8) infection and the disease (12). However, since the publication of the original article describing this correlation, no further evidence has been provided for this hypothesis. Finally, an association between interstitial lung disease and an increased relative abundance of Streptococcus in the oropharyngeal microbiome in CVID was recently identified (13).

The treatment of GLILD mostly consists of immunosuppressive medication, in addition to IgRT and other supportive measures such as physiotherapy. According to the British Lung Foundation/United Kingdom Primary Immunodeficiency Network Consensus Statement, glucocorticoids are the first line of therapy for GLILD (9). Most clinicians agree that azathioprine, mycophenolate mofetil (MMF) and rituximab are second-line choices when glucocorticoids are not effective or when attempting to spare their use (9). Although alternative medication may also be prescribed, there is no consensus about the use of other biologic therapies or disease-modifying anti-rheumatic drugs (DMARDs) (9).

Current GLILD treatment guidelines are based on expert opinion rather than on robust scientific evidence. An objective review of the existing evidence is needed to minimize potential biases associated with expert opinion, and to identify knowledge gaps. Therefore, our aim was to systematically review the existing literature on treatment of GLILD in CVID patients. To the best of our knowledge, this is the first systematic review on that topic.

## Methods

We searched PubMed and EMBASE for publications on treatment of GLILD in CVID patients (last search on March 27th 2020, see **Appendix for Search String**). Articles describing patients with CVID and GLILD who were treated with pharmacological therapy and/or a hematopoietic stem cell transplantation (HSCT) were included. Improvement of disease activity parameters (symptoms, pulmonary function tests and radiological findings) and mortality served as outcomes.

We focused our search on patients with CVID and GLILD. Studies describing patients with monogenetic diseases causing a CVID-like phenotype (such as CTLA-4 haploinsufficiency and LRBA deficiency) were included.

The consensus GLILD definition of the British Lung Foundation/United Kingdom Primary Immunodeficiency Network was used: “GLILD is a distinct clinic-radio-pathological interstitial lung disease occurring in patients with CVID, associated with a lymphocytic infiltrate and/or granuloma in the lung, and in whom other conditions have been considered and where possible excluded” (9). Only articles that reported radiological findings on a CT-scan or histological analysis of biopsies compliant with this definition of GLILD were included.

All non-English articles were excluded for purposes of practicality. Conference abstracts, while read and taken into consideration, were excluded from the review as they were not peer-reviewed.

Two independent investigators (O.L. and B.S.) selected articles on the basis of title and abstract. Blinding of the investigators was achieved by inserting all articles in a common online database (Rayyan), which has a blinding feature and allows each researcher to select articles independently of the other. Ultimately, the selection of articles of each researcher was compared to the other. If there were any selection discrepancies, the articles were discussed until a unanimous decision about in- or exclusion could be made. Data were extracted from the eligible full-text articles using a standardized data extraction sheet. The extracted data were summarized descriptively and reported in tables. We could not conduct meta-analyses because the selected articles contained insufficient quantitative data.

If the use of multiple treatment regimens in one patient was reported, the effect of the treatment regimens was evaluated separately. When escalation or switching of treatment was deemed necessary by the authors, the previous regimen was deemed insufficient. To evaluate the effect of treatment regimens, both qualitative and quantitative assessments of GLILD activity were analyzed. Descriptive improvement of pulmonary function tests, radiological findings and symptoms (e.g. “shortness of breath”, “coughing”) were used for the qualitative evaluation of disease activity. Significant improvement was defined as a relapse-free improvement of at least one of these parameters and no deterioration of the other parameters. Pre- and post-treatment pulmonary function test results were used for the quantitative evaluation of disease activity, and significant improvement of pulmonary function was here defined as a 10% increase in at least one pulmonary function test parameter.

Overall risk of bias of each study was assessed by means of a self-designed tool based on the PRISMA guidelines (14). This tool took into account the quality of the studies (based on the number of patients and controls, and on descriptions of outcomes, medication dosages and follow-up procedures) and possible confounders (smoking, age, comorbidity, and results of genetic testing). Each study was assigned a rating for each of these categories, ‘good’ (+) if the highest quality standard was attained with clear quantitative outcomes, ‘intermediate’ (+/-) if some information was reported but quantitative measures were lacking, and ‘insufficient’ (-) if the information was not reported at all. The overall risk of bias was determined as follows: ‘high risk of bias’ if the study had four or more insufficient or eight or more intermediate judgments; ‘intermediate risk of bias’ if the study was marked insufficient on two to four items or intermediate on four to eight items; and ‘low risk of bias’ if the study had only one insufficient judgment or a maximum of three intermediate judgments.

The level of evidence for each study and the degree of recommendation in clinical practice were determined following the criteria formulated by the Centre for Evidence Based Medicine (15).

## Results

**Figure 1:**
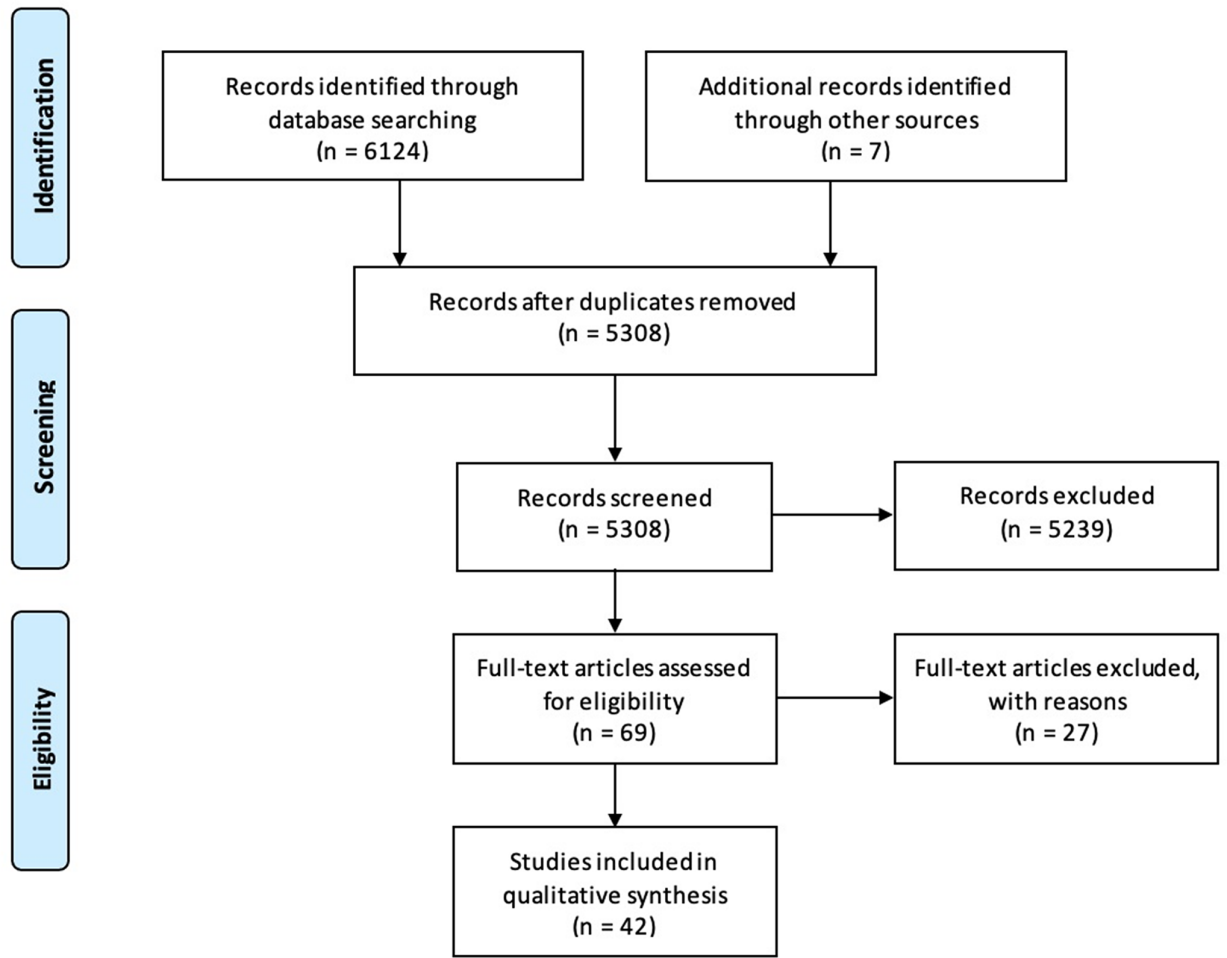
PRISMA flow chart for article inclusion.

**Figure 2:**
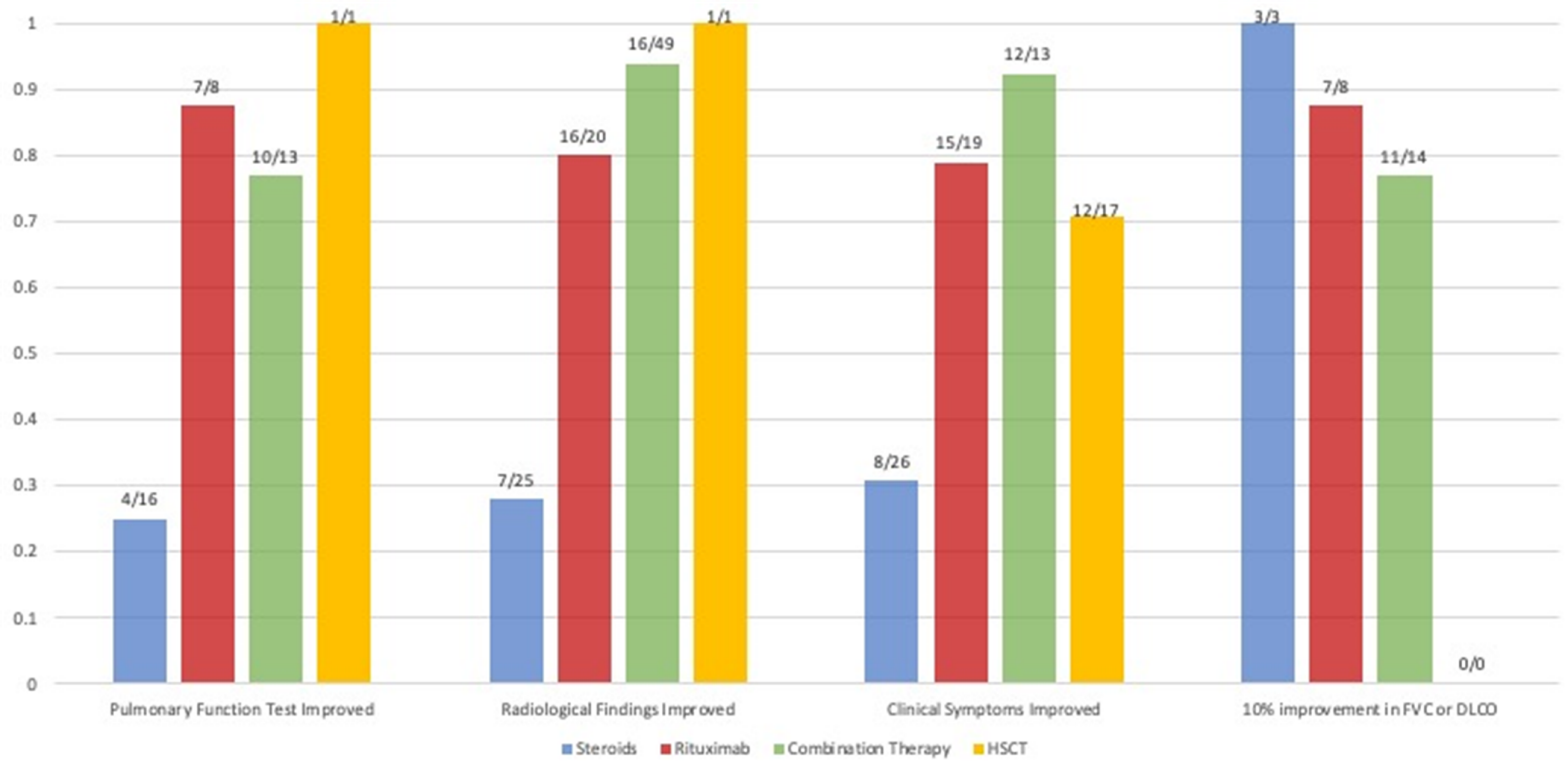
Comparison of the available qualitative and quantitative outcomes of studies that reported on patients (N) treated with steroids, rituximab monotherapy and rituximab combination therapy. The proportion of patients that had a qualitatively reported improvement of pulmonary function tests, radiological findings and the proportion that had a quantitative improvement of their forced vital capacity (FVC) or diffusion capacity of the lung for carbon monoxide (DLCO) of 10% after therapy is shown. Due to a lack of quantitative data, statistics could not be performed.

The search identified 6124 articles on PubMed and EMBASE and seven additional papers *via* snowballing (**Figure 1**). After removal of duplicates, 5304 articles were screened, 65 full-text papers were read, and 42 articles were deemed eligible. 233 patients were described in total. The findings are summarized below, sorted by treatment modality. Qualitative and quantitative lung function findings are shown in **Figure 2**.

There were three papers describing GLILD in patients with B lymphocyte related primary antibody deficiency other than CVID (such as IgA or IgG subclass deficiency, or selective antibody deficiency for polysaccharide antigens). These articles are listed in the **Supplementary Material** (**Table S1**).

### Glucocorticoids

Glucocorticoids have been identified as the first line treatment for GLILD by the British Lung Foundation/United Kingdom Primary Immunodeficiency Network (2017) (9).

Six articles specifically reported on the use of glucocorticoids for the treatment of GLILD in patients with CVID, as shown in **Table 1**. The first report dates back to 1982 and describes the case of a woman who was treated with high-dose prednisone for six weeks. Symptoms initially subsided but relapsed when the medication was tapered (18). Ten additional studies included glucocorticoid treatment as one of several therapies (**Tables 2** and **3**). Five of these reported no effect of glucocorticoids (26, 27, 31, 36–38), one reported relapse after initial remission (29) and four reported treatment success (16, 17, 20, 21). The article by Kanathur et al. is particularly interesting as it describes a case in which glucocorticoids initially failed to have any effect at all but were associated with the resolution of symptoms when paired with splenectomy (19).

**Table 1:** Studies reporting treatment of GLILD in PID with corticosteroids.

Article	Study design	Sample	Intervention	Control	Qualitative outcome	Quantitative outcome
Boujaoude et al. (16)	Case study	32-year-old woman with CVID and GLILD	Prednisone at a dose of 60 mg daily, duration not mentioned	None	Improvement of CS, PFT and RF	FVC: 0.61 L increase ((% predicted increased by 19%), FEV1: 0.48 L increase
Guerrini et al. (17)	Case study	20-year-old woman with CVID and GLILD	Corticosteroids, exact duration not mentioned	None	Improvement of CS and RF	Not mentioned
Kohler et al. (18)	Case study	35-year-old woman with CVID and GLILD	Prednisone at a dose of 60 mg daily for six weeks, after which tapering was initiated	None	Improvement of PFT and RF, relapse when tapering was attempted	FVC: 0.98 L increase (% predicted increased by 28%), FEV1: 0.7 L increase
Kanathur et al. (19)	Case study	67-year-old man with CVID and GLILD	Splenectomy and prednisone at a dose of 60 mg daily for 18 months	None	No effect of prednisone at first, after splenectomy prednisone was continued, resulting in improvement of CS and RF	Not mentioned
Kaufman et al. (20)	Case study	26-year-old woman with CVID and GLILD	Prednisone at a dose of 60 mg daily for a few months, exact duration not mentioned	None	Improvement of PFT and RF	FVC: 0.08 L increase (% predicted increased by 2%) FEV1: 0.01 L increase (no change in % predicted)), DLCO: 2.9 ml/mm/mmHg (% of predicted increased by 13%)
Wislez et al. (21)	Case study	68-year old woman with CVID and GLILD	Prednisone at a dose of 0.75 mg per kg daily, then tapering to 5 mg daily over the course of two months and stopping completely eight months later.	None	Improvement of CS and RF, but relapse upon interruption of glucocorticoids. Improvement of symptoms upon reintroduction of glucocorticoids.	Not mentioned

CVID, common variable immunodeficiency; CS, clinical symptoms; DLCO, diffusing capacity; FVC, forced vital capacity; FEV1, forced expiratory volume in 1 second; GLILD, granulomatous-lymphocytic interstitial disease; MMF, mycophenolate mofetil; MTX, methotrexate; PFT, pulmonary function tests; RAG, recombination-activating gene; RF, radiological findings.

**Table 2:** Studies reporting treatment of GLILD in antibody deficiencies with various immunosuppressants.

Article	Study design	Sample	Intervention	Control	Qualitative outcome	Quantitative outcome
Ardenitz et al. (22)	Prospective follow up cohort study	37 patients with CVID and granulomatous disease, of which 20 also had GLILD	Splenectomy was performed in nine patients, 29 patients were given glucocorticoids, with or without other therapies, 10 subjects were also given one or more additional immune suppressants: hydroxychloroquine (five subjects), cyclosporine (three subjects), azathioprine (two subjects), methotrexate (two subjects), infliximab (one subject), and etanercept (one subject). One patients was administered rituximab. Five patients received no treatment. Duration of treatments varied. Treatment of 13 patients with GLILD was specifically reported. Patient 04: prednisone and hydroxychloroquine Patient 08: cyclosporine at a dose pf 100 mg twice daily, years of prednisone, IV glucocorticoids Patients 11: monthly oral and IV glucocorticoids Patient 14: chronic prednisone at a dose of 20 mg daily Patient 20: oral prednisone for 12 months Patients 21 oral prednisone for 12 months Patient 24: infliximab, hydroxychloroquine at a dose of 200 mg twice daily for 15 years Patient 28: MTX at a dose of 7.5 mg weekly for 12 months, hydroxychloroquine at a dose of 200 mg twice daily for five years Patient 34: years of prednisone, hydroxycholoroquine Patient 35: years of steroids at a dose of 10 mg every two days Patient 36: oral steroids at a dose of 5 mg daily for one week, COX2 inhibitors	Patients with same disease received different treatments	Outcomes were not reported for single patients. 10 (28.5%) patients died (seven of pulmonary complications and at least five with GLILD), rituximab led to resolution of autoimmunity, unclear how other drugs were effective	Not mentioned
Boursiquot et al. (23)	Prospective follow up cohort study	59 patients with CVID of which 30 also had GLILD	25 treatment regimens were noted. Oral corticosteroids were administered to 13 patients for a median of 18 months, six received cyclophosphamide for a median of six months, hydroxychloroquine was used in four cases for a median of 13.5 months, rituximab in three for a median of six months. MTX for a median of 38 months, thalidomide for a median of two months, infliximab and azathioprine were each used in two patients for a median of 31 and 18 months respectively. Cyclosporine, Interferon alpha, MMF and sirolimus were used in one patient each, for a median of 12, six, 20 and 12 months	31 patients with CVID who did not receive any treatment	Complete remission was obtained in three patients who were treated with corticosteroids, one who was treated with MTX and one who was treated with cyclophosphamide. 10 patients had a partial response and 10 had no effect at all	Not mentioned
Bouvry et al. (24)	Prospective follow up cohort study	20 patients with CVID and GLILD	17 patients received IVIg, 15 corticosteroids, three others not specified immunosuppressants and two hydroxychloroquine, duration not specified	60 patients with sarcoidosis	Six of the patients with CVID and GLILD died, all of the patients with sarcoidosis were still alive	Not mentioned
Bucciol et al. (25)	Case study	Three patients with CVID and GLILD: 23-year-old man, 18-year-old man and 4-year-old girl	Corticosteroids, duration not specified MMF, duration not specified	None	Resistance to steroids or relapse despite steroids. Stabilization of CS and improvement of RF after MMF administration	Pt 1; FVC: (% predicted decreased by 7%, FEV1: (% predicted decreased by 4%. Pt 2: Pre-treatment data not mentioned, FVC after treatment 60% of predicted FEV1 after treatment 68% of predicted Pt 3: not mentioned
Cha et al. (26)	Prospective follow-up cohort study	15 patients with various underlaying diseases (one had CVID)and GLILD)	Corticosteroids, MTX, colchicine, azathioprine, cyclophosphamide and cyclosporin. Patient with GLILD: corticosteroids and MTX, later switched to cyclosporin, duration not mentioned	None	Patient with CVID: still alive, no effect of corticosteroids and MTX, improvement of CS and PFT when switched to cyclosporin	Not mentioned
Davies et al. (27)	Case study	34-year-old woman CVID and GLILD	Prednisone at a dose of 40 mg daily Cyclosporin at a dose of 125 mg daily	None	No effect of prednisone, improvement of CS and RF on cyclosporin A	FVC: 0.71 L increase ((% predicted increased by 30%), FEV1: 0.6 L increase
Deya-Martinez	Case study	2 patients (12-year-old boy with CVID and GLILD and 16-year-old girl with Kabuki syndrome and GLILD)	Pt 1: rituximab at a dose of 375 mg per m2 weekly for 4 weeks twice. MMF and sirolimus at dose of 2.5 mg/m2 daily, duration not specified Pt 2: sirolimus, duration not specified	None	Pt 1: Good effect of rituximab initially, but relapse six months after treatment. Improvement of with MMF and sirolimus. Pt 2: Improvement of RF with sirolimus	Not mentioned.
Franxman et al. (28)	Case series	3 patients with CVID and GLILD (14-year-old female, 55-year-old female and a 16-year-old male)	Pt 1: Corticosteroids and MMF, dose and duration not specified. Infliximab 5 mg/kg every 4 weeks for 4 months Pt 2: Corticosteroids and plaquenil, dose and duration not specified. Infliximab 5 mg/kg every 4 weeks for 6 months Pt 3: Corticosteroids, dose and duration not specified. Infliximab 5 mg/kg every 4 weeks for 5 months		Pt 1: No effect of corticosteroids, after initiation of infliximab steroids could be tapered and there was improvement of CS, PFT and RF. Pt 2: Decline of RF PFT and CS during corticosteroid therapy. Improvement of CS & PFT. Discontinuation of treatment due to possibly treatment related skin lesions. Pt 3: Relapse upon tapering of steroids. Improvement of CS & PFT and successful taper of steroids after infliximab introduction	Pt 1; FVC: increased by 22%, FEV1: increased by 20% Pt 2; FVC: increased by 6%, DLCO: increased by 33%. Pt 3; FVC: increased by 35%
Sacco et al. (29)	Case study	Six-year-old girl with CVID and GLILD	Corticosteroids at a dose of 2 mg per kg daily for two weeks, after which tapering was started. A dose of 0.75 mg per kg daily was maintained for three years, until it was further tapered to 0.17 mg per kg per day. Azathioprine at a dose of 1.5 mg daily, for the duration of three years, after which the dose was tapered to 0.75 mg per kg per day	None	Improvement of clinical symptoms and RF with corticosteroids only, but relapse when tapering. Addition of azathioprine stabilised situation	Not mentioned
Tashtoush et al. (30)	Case study	51-year-old patient with CVID and GLILD	Prednisone at a dose of 0.5 mg per kg daily for 3 months MMF at a dose of 1000 mg daily for nine months	None	Improvement of CS and RF after 3 months	Not mentioned
Thatayatikom et al. (31)	Case study	22-year-old man with CVID and GLILD	High-dose methylprednisolone Infliximab at a dose of 10 mg daily for six weeks. After relapse treatment with infliximab was re-initiated at a dose of 5 mg daily for nine months	None	No effect of methylprednisolone, improvement after addition of infliximab, then relapse with interruption of treatment. Again, improvement of CS and RF after therapy re-initiation	Not mentioned

CVID, common variable immunodeficiency; CS, clinical symptoms; DLCO, diffusing capacity; FVC, forced vital capacity, FEV1, forced expiratory volume in 1 second; GLILD, granulomatous-lymphocytic interstitial disease; MMF, mycophenolate mofetil; MTX, methotrexate; PFT, pulmonary function tests; RAG, recombination-activating gene; RF, radiological findings.

**Table 3:** Studies reporting treatment of GLILD in PID with rituximab.

Article	Study design	Sample	Intervention	Control	Qualitative outcome	Quantitative outcome
Arraya et al. (32)	Case report	57-year-old female with CVID and GLILD	Rituximab at a dose of 375 mg/m2 weekly for four cycles. Three cycles were used for induction, a yearly cycle was used for maintenance for 8 years.	None	Improvement of RF	Not mentioned
Ceserer et al. (33)	Case series	Three patients with CVID and GLILD (38- and 56-year-old women, 44-year-old man)	Rituximab at a dose of 375 mg/m2 weekly for four cycles. At total of 16 infusions was given	None	Improvement of CS, PFT and RF	Pt 1; FVC: 0.37 L increase ((% predicted increased by 11%), DLCO: 0.6 ml/mm/mmHg increase ((% predicted increased by 8%), FEV1: 3.04 L increase ((% predicted increased by 38%) Pt 2; FVC: 0.36 L increase ((% predicted increased by 24%), DLCO: 0.4 ml/mm/mmHg increase ((% predicted increased by 7%), FEV1: 0.19 L increase ((% predicted increased by 12%) Pt 3: FVC: 0.25 L decrease ((% predicted decreased by 4%), DLCO: 0.9 ml/mm/mmHg increase ((% predicted increased by 9%), FEV1: 0.36 L decrease ((% predicted decreased by 7%).
Maglione et al. (10)	Prospective cohort study	11 patients with CVID and progressive GLILD	Rituximab at a dose of 375 mg/m2 weekly for four cycles	44 patients with CVID but no GLILD, 14 patients with CVID and stable GLILD and four patients with CVID and progressive GLILD	Improvement of CS and RF. Relapse of 4 patients.	Not mentioned
Ng et al. (34)	Case study	Two patients with CVID and GLILD (36-year-old man and 33-year-old woman)	Corticosteroids, duration not specified Rituximab at a dose of 375 mg/m2 weekly for four cycles with a four- to six-month interval. A total of 16 infusions was given	None	Corticosteroids led to short-lived improvement of CS, rituximab led to improvement of CS and RF	Not mentioned
Tessarin et al. (35)	Case study	37-year-old woman with CVID and GLILD	Rituximab at a dose of 375 mg/m2 every four weeks, weekly for four cycles with a four to six month interval	None	Improvement of CS and RF	Not mentioned
Vitale et al. (36)	Case study	37-year-old woman with CVID and GLILD	High-dose corticosteroids, duration not specified Rituximab at a dose of 375 mg/m2 every four weeks, weekly for four cycles with a four to six month interval	None	Corticosteroids had no direct effect, addition of rituximab led to improvement of CS, PFT and RF	Not mentioned
Zdziarsky and Gamian (37)	Case study	25-year-old woman with CVID and GLILD	Methylprednisone at a dose of up to 50 mg daily, duration not specified Rituximab at a dose of 150 mg/m2 weekly for six cycles and later at a dose of 375 mg/m2 every 21 days for four cycles with a six-month remission interval	None	No effect of corticosteroids, improvement after first underdosed cycle of rituximab followed by relapse, improvement of CS and RF after second cycle of rituximab	FVC: 1.21 L increase

CVID, common variable immunodeficiency; CS, clinical symptoms; DLCO, diffusing capacity; FVC, forced vital capacity, FEV1, forced expiratory volume in 1 second; GLILD, granulomatous-lymphocytic interstitial disease; PFT, pulmonary function tests; RF, radiological findings.

### Conventional Disease Modifying Anti Rheumatic Drugs (DMARDs)

Besides glucocorticoids, other immunosuppressants for the treatment of GLILD have been evaluated (**Table 2**). Examples encountered in the literature included methotrexate (MTX), cyclophosphamide, mycophenolate (MMF), azathioprine, cyclosporin, hydroxychloroquine, tacrolimus and sirolimus.

Boursiquot et al. assessed the efficacy of both MTX and cyclophosphamide in the treatment of GLILD. The researchers prospectively followed 59 patients with CVID, of whom 30 had GLILD. Different treatment regimens were initiated in 25 patients with CVID and GLILD (**Table 2**). Complete remission was obtained in three (out of 13) patients who were treated with glucocorticoids, one (out of one) who was treated with MTX and one (out of five) who was treated with cyclophosphamide. Ten patients had a partial response and the remainder showed no effect at all (23).

Other articles reported the use of MMF for the treatment of GLILD. Bucciol et al. described three patients with GLILD. Glucocorticoids were ineffective, but a switch to MMF resulted in stabilization of symptoms and improvement of clinical and radiologic findings in all three cases (25). More evidence was provided by Tashtoush et al., who published a case report about a 51-year old woman with CVID and GLILD. This patient achieved remission after induction therapy with glucocorticoids for 3 months and MMF maintenance therapy for 9 months (30).

As emerged from the Delphi Study of the British Lung Foundation/United Kingdom Primary Immunodeficiency Network, azathioprine is another drug that is often used for the treatment of GLILD. An article dating back to 1996 by Sacco et al. reported the case of a six-year-old girl with CVID and severe GLILD. The patient was treated with glucocorticoids with good effect, but tapering of the medication resulted in disease relapse. This prompted the physicians to add azathioprine, which halted disease progression. The combination of prednisone and azathioprine was maintained for three years, after which they were tapered to 5 mg every other day and 0.75 mg per kg daily, respectively (29).

Albeit less frequently reported, several articles describe the use of cyclosporine for the treatment of GLILD. Davies et al. reported the case of a 34-year old woman with CVID and GLILD who responded well to glucocorticoid therapy, but had recurrent relapses after tapering. The patient was eventually treated with cyclosporine, with good effect (27). Similar results were observed by Cha et al.: a patient with CVID and concomitant GLILD was initially treated with glucocorticoids, but achieved disease remission only when therapy was switched to cyclosporin (26).

Deya-Martinez et al. showed that the immunosuppressant sirolimus can be useful in the treatment of GLILD. A boy with CVID and GLILD, who had been previously treated with rituximab and who had relapsed, was switched to sirolimus monotherapy and achieved remission of symptoms (39).

Two articles reported the use of DMARDs for the treatment of GLILD in relatively large patient series. Both papers described variable regimens of multiple drugs, without mentioning the outcomes.

Ardeniz described the long-term follow up of a group of 37 patients with CVID and granulomatous disease, of which 20 patients had GLILD. Patients were treated with a different combination of drugs, including glucocorticoids, cyclosporine, hydroxychloroquine, infliximab, etanercept and rituximab. Outcomes were not clearly reported. Over the follow-up period of 25 years, 10 of the 37 patients included in the study died. Of those, at least five had GLILD (22).

Bouvry compared outcomes of CVID patients with GLILD with those of patients with sarcoidosis. Patients were treated with different immunosuppressants over the course of the study. Results were not clearly reported, the main difference between the two groups was that patients with CVID and GLILD had worse outcomes than those with sarcoidosis (24).

### Biologicals

Biologicals, also known as biological medicinal products, are drugs which are (partially) produced by living organisms by means of recombinant DNA technologies (40). For GLILD specifically, infliximab, rituximab and abatacept have been used.

### Infliximab

Infliximab is a monoclonal antibody that binds to TNFα and blocks signaling, thus interfering with a central mechanism of inflammation (41). Thatayatikom et al. reported a 22-year-old man with CVID and life-threatening GLILD, who was first unsuccessfully treated with glucocorticoids, but achieved remission after treatment with infliximab for nine months (31). Additionally, Franxman, Howe & Baker described three patients who all showed remission of GLILD on CT scan and pulmonary function tests, after 4 months, 8 months and 5 months of treatment, respectively (28).

### Rituximab

Rituximab is a monoclonal antibody that depletes B-cells, by binding to CD20 molecules on their surface (42). Seven studies focused on rituximab monotherapy for GLILD (**Table 3**). Arraya, Cereser, Ng and Tessarin all reported cases of patients with CVID and GLILD who were successfully treated with rituximab monotherapy (at a dose of 375 mg/m2 weekly for four weeks) (32–35). Maglione et al. followed 73 patients for 18 months: 44 patients had CVID only, 14 had concomitant stable GLILD, and 15 had concomitant progressive GLILD. 11 of the 15 patients with progressive GLILD were treated with rituximab at a dose of 375 mg/m2 weekly for four weeks: all experienced stabilization or improvement of disease activity, however four relapsed 18 months after completion of therapy (10).

Of particular interest is the study by Zdziarsky and Gamian’s, describing a 25-year old woman with CVID and GLILD who was treated with rituximab monotherapy at a relatively low dose of 150 mg/m2 weekly for six weeks because of risk of infection (37). This resulted in incomplete remission of clinical symptoms, and the patient relapsed six months later. Treatment with rituximab was repeated, this time at a dose of 375 mg/m2, resulting in complete remission for a period of 30 months.

### Combination Chemotherapy With Rituximab and Azathioprine

Eight studies evaluated combination chemotherapy with rituximab and azathioprine (**Table 4**). The rationale behind this combination chemotherapy is that B- and T-lymphocytes are targeted simultaneously (38). Chase and colleagues were the first ones to pioneer this approach. They performed a longitudinal prospective cohort study in which they followed seven patients with CVID and GLILD, who were treated with intravenous rituximab and oral azathioprine for 18 months. All patients experienced some degree of improvement in radiological findings (38). These results were confirmed by Pathria, Routes, Limsuwat and Tillman, who reported successful treatment of patients with CVID and GLILD with combination chemotherapy (44–46, 49). Vitale et al., reported successful addition of combination therapy with rituximab to glucocorticoid treatment in a 17-year old patient with CVID and GLILD after initial unresponsiveness to glucocorticoid monotherapy (36). Jolles’ and Sood’s articles showed that azathioprine can be replaced by other drugs with similar mechanisms of action. For example, Jolles et al. described a 51-year old woman with CVID and GLILD treated with a combination of rituximab and MMF, because of intolerance of azathioprine. Five months into treatment, the patient experienced an improvement of symptoms, alongside better pulmonary function and radiologic results (43). Sood et al. reported an improvement of GLILD related symptoms in the case of a 16-year old boy with 22q.11 deletion syndrome who was treated with rituximab and 6-mercaptopurine (48). One additional article by Verbsky et al. was added to the review despite its publishing date (June 2020) being after the last literature search (March 2020). We choose to mention this article, because the planned publication of the paper was known to the authors at the time of the literature search and, most importantly, because its results are highly relevant for this systematic review. The authors performed a retrospective chart review of 39 patients with CVID and GLILD who were treated with a combination of rituximab and azathioprine or rituximab and MMF. The median follow-up period was four years. 37 patients were included in the final analysis and of those 34 (92%) experienced an improvement of GLILD-related parameters. 27 patients (73%) experienced sustained remission, whereas nine patients (24%) relapsed after a median of 3.2 months. Of those relapsing, two patients died of septicemia and respiratory failure, respectively (47).

**Table 4:** Studies reporting treatment of GLILD in antibody deficiencies with combination chemotherapy.

Article	Study design	Sample	Intervention	Control	Qualitative outcome	Quantitative outcome
Chase et al. (38)	Prospective follow-up cohort study	Seven patients with CVID and GLILD	Five patients received corticosteroids Rituximab at a dose of 375 mg/m2 weekly for four cycles with a four to six month interval. A total of 12-16 infusions was given Azathioprine at a dose of 1-2 mg per kg for 18 months	None	No effect of corticosteroids, combination chemotherapy led to improvement of CS and RF	Pt 1; FVC: 0.52 L increase ((% predicted increased by 9%), FEV1: 0.3 L increase ((% predicted increased by 9%), DLCO 6.89 increase ((% predicted increased by 27%).Pt 2; FVC: 0.4 L increase ((% predicted increased by 13%), FEV1: 0.11 L increase ((% predicted increased by 6%), DLCO after treatment 22.1 (98% of predicted).Pt 3; FVC: 0.11 L increase ((% predicted increased by 2%), FEV1: 0.09 L increase ((% predicted increased by 2%), DLCO 5.3 decrease ((% predicted decreased by 19%).Pt 4; FVC: 0.4 L increase ((% predicted increased by 5%), FEV1 0.4 L increase ((% predicted increased by 7%), DLCO 2.9 increase ((% predicted increased by 9%).Pt 5; FVC: 0.22 L decrease ((% predicted decreased by 4%), FEV1: 0.14 L decrease ((% predicted decreased by 2%), DLCO: 0.51 increase ((% predicted increased by 3%).Pt 6; FVC: 1.22 L increase ((% predicted increased by 33%), FEV1: 0.97 L increase ((% predicted increased by 31%), DLCO after treatment 19.00 (76% of predicted).Pt 7; FVC: 0.73 L (18% of predicted), FEV1 0.49 L (16% of predicted), DLCO 6.6 increase (20% of predicted).
Jolles et al. (43)	Case study	51-year-old woman with CVID and GLILD	Rituximab in two doses of 1g MMF for seven months	None	Improvement of PFT and RF	FVC: % predicted increased by12.5%, DLCO: % predicted increased by 10.9%
Limsuwat et al. (44)	Case study	56-year-old man with CVID and GLILD	Rituximab at a dose of 375 mg/m2 for four weeks, followed by azathioprine 200 mg/d	None	Improvement of CS, CT and PFT	FVC: 1.0 L increase (53% increase), FEV1: 0.45 L increase (46% increase)
Pathria et al. (45)	Case study	61-year old woman with CVID and GLILD	Rituximab at a dose of 375 mg/m2 was initiated. A total of four infusions were given Azathioprine at a dose of 0.75 per kg, which was increased to 1.5 mg per kg after two months	None	Improvement of CS and RF	Not mentioned
Routes and Verbsky (46)	Case study	17-year old girl with CVID and GLILD	Corticosteroids for other auto-immune manifestations Rituximab and azathioprine (dose not mentioned)	None	Improvement of PFT & RF	Not mentioned
Verbsky et al. (47)	Retrospective cohort study	37 patients with CVID and GLILD	One patient received glucocorticoids prior to combination chemotherapy (dose not mentioned) Rituximab at a dose of 375 mg/m2 weekly for four cycles with a four to six-month interval. A total of 16 infusions was given Azathioprine at a dose of 1-2 mg per kg daily or MMF at a dose of 250-1000 mg twice daily for a median of 16 months		Glucocorticoids had no effect. Improvement of RF in 34/37 (92%) after combination chemotherapy. Remission was maintained in 27 patients, 9 had relapses after a median of 3.2 years, one patient underwent lung transplantation. Two patients eventually died, one of septicemia seven months after completion of treatment and the other of respiratory failure (not mentioned at which timepoint after treatment)	At baseline, FEV1 and FVC were normal in 16 (41%) patients, restrictive in 17 (44%), obstructive in 2 (%%) and mixed obstructive-restrictive in 4 (10%). 29 GLILD had DLCO measurements, 14 were normal (48%)*
Sood et al. (48)	Case study	16-year old boy with 22q.11 deletion syndrome, CVID and GLILD	Corticosteroids for other auto-immune manifestations Rituximab at a dose of 375 mg/m2 6-Mercaptopurine at a dose of 0.5 mg per kg three times weekly	None	Improvement of CS	Not mentioned
Tillman et al. (49)	Case study	13-year-old girl with CVID and GLILD	Rituximab at a dose of 375 mg/m2 weekly for four cycles Azathioprine at a dose of 50 mg once daily for 18 months	None	Improvement of CS and RF	FVC: increase of 64% of predicted FEV1: increase of 49% of predicted
Vitale et al. (36)	Case study	17-year-old boy with CVID and GLILD and intracranial lymphoproliferative lesions	High-dose corticosteroids Rituximab at a dose of 375 mg/m2 weekly for four cycles with a four to six-month interval. A total of 16 infusions was given Azathioprine at a dose of 1.7 mg per kg for 18 months	None	Corticosteroids had no effect, rituximab led to improvement of CS and RF with resolution of intracranial lesions	FVC: 0.62 L increase, FEV1: 0.54 L decrease

*In the paper by Verbsky et al. (47), the total number of patients included are 39, the total number of patients treated with combination chemotherapy were 27.

CVID, common variable immunodeficiency; CS, clinical symptoms; DLCO, diffusing capacity; FVC, forced vital capacity; FEV1, forced expiratory volume in 1 second; GLILD, granulomatous-lymphocytic interstitial disease; MMF, mycophenolate mofetil; PFT, pulmonary function tests; RF, radiological findings.

### Abatacept

CTLA-4 haploinsufficiency and LRBA deficiency result in a phenotype similar to CVID with severe immunodeficiency, lymphoproliferation and autoimmunity. In the physiological state, T lymphocyte responses are regulated by binding of the B7 ligand to CTLA-4 thus blocking T-cell activation, whereas LRBA is involved in intracellular trafficking and, among others, preserves CTLA-4 from degradation (50, 51), causing excessive immune activation. Abatacept consists of the Fc region of immunoglobulin IgG1 fused to CTLA-4 (52) and thus serves as a CTLA-4 fusion protein preventing excessive T lymphocyte proliferation in patients with CTLA-4 haploinsufficiency and LRBA deficiency.

A total of three articles described the use of abatacept for the treatment of GLILD (**Table 5**). Schwab and colleagues performed a longitudinal prospective cohort study in which they followed 133 patients with CTLA-4 haploinsufficiency. Of these, two patients who presented with GLILD treated with abatacept experienced improvement of both clinical symptoms and radiologic findings (51).

**Table 5:** Studies reporting treatment of GLILD in PID with abatacept.

Article	Study design	Sample	Intervention	Control	Qualitative outcome	Quantitative outcome
Kostel Bal et al. (53)	Case study	7 patients with LBRA deficiency, one of which had concomitant GLILD (12-year-old boy)	Abatacept at a dose of 20 mg per kg every two weeks, duration not specified	None	Improvement of RF	Not mentioned
Lo et al. (54)	Prospective follow-up cohort study	Nine patients with LBRA deficiency, three of whom also had GLILD	Corticosteroids and MMF, duration not specified Abatacept in different doses: 20 mg per kg every two weeks, 20 mg per kg every four weeks, 30 mg per kg monthly for six months	None	Disease progression despite treatment with corticosteroids and MMF Improvement in clinical symptoms, PFT and RF	Pt 1: FVC: % predicted increased by 30-40%, FEV1: % predicted increased 35%, DLCO % predicted increased by 35%. Pt 3: FVC: % predicted increased by 50% of predicted, FV1: % predicted increased by 40%, DLCO % predicted increased by 50%.
Schwab et al. (51)	Prospective follow-up cohort study	90 CTLA4 mutation carriers, of which 32 with GLILD	Abatacept was administered to 14 patients, duration not specified	43 unaffected mutation carriers	Six of the patients treated with abatacept experienced improvement of symptoms (two who had GLILD had resolution of lymphoproliferative lesions)	Not mentioned

Lo and colleagues reported three patients with LRBA deficiency and GLILD, who experienced significant improvements in lung function and radiological findings after treatment with abatacept (54). Bal replicated these results, findings abatacept to be useful in the treatment of GLILD in a 12-year old boy with LRBA deficiency (53).

### Hematopoietic Stem Cell Transplantation

HSCT holds the promise of being a definitive treatment for GLILD as it can correct the underlying immunodeficiency and the associated GLILD instead of just alleviating GLILD related symptoms. However, it is associated with considerable risks, including Graft versus Host Disease (GvHD) and serious infections, both associated with considerable morbidity. This risk is likely higher in those with established structural lung disease.

Five studies reported on HSCT for CVID patients with associated GLILD (**Table 6**). Wehr followed 25 patients with CVID who underwent HSCT. Five patients had GLILD: four experienced an improvement of the CVID-related complications; one died 104 days after transplantation due to acute GvHD and infectious complications (60). Wehr’s papers also includes four patients which were discussed in Rizzi’s publication in 2011 (56). Hartono published the case of a 23- year-old woman who presented with a CVID-like phenotype due to a STAT1 gain-of-function mutation and GLILD: after HSCT there was an improvement of radiologic findings (55). Mixed outcomes were reported by both Seidel and Tesc. Seidel and colleagues performed an international survey and collected information about 12 patients with CVID-like disease due to underlying LRBA deficiency (seven of whom also had GLILD), who underwent HSCT. Four patients went into partial remission, whereas three of them died (57). Tesch published a prospective follow-up study of 76 patients with LRBA deficiency, of which 24 underwent HSCT. Of these 24 patients, 17 of the 24 patients survived and all of the seven patients with concomitant GLILD experienced an improvement of GLILD related symptoms. Two patients who did not have GLILD before HSCT, developed the disease after the procedure (59).

**Table 6:** Studies reporting treatment of GLILD in antibody deficiencies with HSCT.

Article	Study design	Sample	Control	Donor	Conditioning*	GVHD prophylaxis	Outcome (GLILD)	Outcome (Survival)
Hartono et al. (55)	Case study	23-year old girl with STAT1 mutation and GLILD	None	MUD	Not mentioned	Steroids	Improvement of radiological findings	Patient still alive day +522 post-transplant
Rizzi et al. (56)	Case study	One patient with CVID and GLILD	None	Patient 004: MUD	Patient 004: RIC^1^	CsA	Subjective improvement of PFT and reduction of steroids use	Patient with GLILD survived
Seidel et al. (57)	Prospective follow up cohort study	12 patients with LBRA deficiency of which seven also had GLILD	None	Patient 001: MFD Patient 002: MSD Patient 004: MUD Patient 006: MMFD Patient 008: MUD Patient 010: MUD Patient 01: MSD	Patient 001 RIC^2^ Patitent 002 RIC^3^ Patient 004 RIC^4^ Patitent 006 RIC^5^ Patient 008 RIC^6^ Patient 010 RIC^7^ Patient 011 RIC^8^	Not mentioned	Patients 002 and 010 with GLILD had complete remission (no symptoms and no need for medication), patient 001 with GLILD had good partial remission (some symptoms but no need for medication), patient 011 with GILD had partial remission (improvement of symptoms but still need for medication)	Overall survival was 67% (8/12). Patient 004, 006 and 008 with GLILD died three and two months post procedure
Slatter et al. (58)	Prospective follow up cohort study	Two patients with CTLA4 deficiency and GLILD	None	MUD	Not mentioned	Five patients (1, 2, 5, 6, and 8) CsA and MMF for GVHD. Three (3, 4, and 7) had CsA alone, CsA and MMF, or MTX and tacrolimus. Patient 6 had prednisolone, sirolimus, and belatacept until 8 days before transplant	Improvement of symptoms, tapering of immunosuppressive medication.	Six patients are still alive (two patients with GLILD fall in this group and are alive and well at 4 months and 4 years post-transplantation), two died of GvHD and DKA, respectively
Tesch et al. (59)	Prospective follow up cohort study	76 patients with LBRA deficiency of which 24 underwent HSCT and 17 had GLILD	Patients who did not undergo HSCT	Patient 001: MMUD Patient 002: MSD Patient 003: MSD Patient 004: MSD Patient 005: MFD Patient 007: MSD Patient 010: MUD Patient 014: MSD	Patient 001 RIC^9^ Patient 002 MAC^10^ Patient 003 RIC^11^ Patient 004 RIC^12^ Patient 005 RIC^13^ Patient 007 RIC^14^ Patient 010 RIC^15^ Patient 014 RIC^16^	Not mentioned	Of the eight patients with GLILD, five are in complete remission, two are in partial remission with still some symptoms of GLILD. Of the 24 patients undergoing HSCT, two developed GLILD after the procedure	Overall survival was 70.8% (17/24)
Wehr et al. (60)	Prospective follow-up cohort	Two patients with CVID and GLILD	None	Patient 004: MUD Patient 029: MUD	Patient 004: RIC^17^ Patient 028: MAC^18^	Patient 004: CsA Patient 028: CsA, sirolimus, MMF, corticosteroids	Patient 004: not mentioned Patient 028: deceased	Patient 028 died 104 days after procedure of aGvHD and infectious complications

Ale: Alemtuzumab; ATG: anti-thymocyte globulin; Bu: Busulfan; CsA: Cyclosporin A; CP: cyclophosphamide;Flu: Fludarabine; MAC: myeloablative conditioning; Mel: Melphalan; MFD: matched family donor;MMFD: mismatched family donor; MMUD: mismatched unrelated donor; MSD: matched sibling donor; MUD: matched unrelated donor; RIC: reduced intensity conditioning.

Conditioning*: only conditioning regimens for patients with PADs were reported. ^1^Flu, Mel and Ale,^2^Flu, ATG, Treo, ^3^Flu, ATG ,^4^Flu, ATG, Treo, Thiotepa,^5^Flu, ATG, Thiotepa, Mel, ^6^Flu, ATG, Mel, ^7^Flu, ATG, Thiotepa, ^8^Flu, ATG, Treo, ^9^Fly, ATG, Mel, ^10^CP, Bu, ^11^Flu, ATG, Mel, ^12^Flu, ATG, Mel, ^13^Flu, ATG, Treo, Thiotepa, ^14^Flu, ATG, Treo, Thiotepa, ^15^Flu, ATG, Treo, Thiotepa, ^16^Flu, ATG, Mel, ^17^Flu and Mel, ^18^Bu and Flu,

### Quality of Studies and Level of Evidence

All studies had an overall intermediate or high risk of bias (**Table 7**). This was largely due to the small sample sizes and lack of controls. Outcomes were mostly reported qualitatively, with few data about pulmonary function tests and a lack of standardized CT evaluation. The duration of follow-up was typically limited, meaning that long-term outcomes of patients remained uncertain. As far as confounders are concerned, smoking status was not always reported. Finally, genetic testing for CTLA-4 haploinsufficiency and LRBA deficiency only became available as of 2012, meaning that older articles could not make this additional distinction.

**Table 7:** Quality of studies analyzing treatment for GLILD in primary antibody deficiencies.

	Quality of the study					Confounders				
Article	Study Design	Controls	Outcome	Follow-up	Dose	Smoking	Age	Co-morbidities	Genetic testing	Overall risk of bias
Arraya et al.	–	–	+/-	+	+	–	+	+	–	High
Ardenitz et al.	+	+	–	+	–	–	+	–	–	High
Boujaoude et al.	–	–	+	–	+	+	+	+	–	High
Boursiquot et al.	+	+	+/-	+	+/-	–	+/-	+/-	–	High
Bouvry et al.	+	+/-	–	–	–	–	+	–	–	High
Bucciol et al.	–	–	+/-	+	–	–	+	+	–	High
Ceserer et al.	–	–	+/-	+	+	–	+	–	–	High
Cha et al.	+	+/-	+/-	+	–	+	+	+	–	Intermediate
Chase et al.	+/-	–	+	+/-	+	–	+	–	+	High
Davies et al.	–	–	+	+	+	+ (non smoker)	+	+	–	Intermediate
Deya-Martinez et al.	–	–	+/-	+/-	+	-(children)	+	+	+	High
Franxman et al.	+/-	+/-	+	–	+	–	+	+	–	High
Guerrini et al.	–	–	+/-	–	–	–	+	+	–	High
Hartono et al.	–	–	+/-	+	NA	–	+	+	+	Intermediate
Jolles et al.	–	–	+/-	+	+	–	+	+	–	High
Kanathur et al.	–	–	+/-	+	+	+	+	+	–	Intermediate
Kaufman et al.	–	–	+	+/-	+	–	+	+	–	High
Kohler et al.	–	–	+	+	+	–	+	+	–	High
Kostel Bal et al.	–	–	+/-	–	+	–	+	+	+	High
Limsuwat et al.	–	–	+	+/-	+	+	+	+	–	Intermediate
Lo et al.	+/-	+/-	+/-	+	+	–	+	+	+	Intermediate
Maglione et al. (8)	–	+	+/-	–	+	–	+	+	–	High
Maglione et al. (10)	+	+	+/-	+	+	–	+	+	–	Intermediate
Ng et al.	–	–	+/-	+	+	–	+	+	–	High
Pathria et al.	–	–	+/-	–	+	+	+	+	–	High
Rizzi et al.	–	–	+/-	+	NA	–	+	+	–	High
Routes & Verbsky	–	–	+/-	–	–	–	+	+	–	High
Sacco et al.	–	–	+/-	+	+	–	+	+	–	High
Schwab et al.	–	+/-	+/-	–	–	–	+	+	+	High
Seidel et al.	+/-	–	+/-	+	NA	–	+	+	+	Intermediate
Slatter et al.	+/-	–	+/-	–	NA	–	+	+	+/-	High
Sood et al.	–	–	+/-	+/-	+	–	+	+	+	Intermediate
Tashtoush et al.	–	–	+/-	+/-	+	+ (non smoker)	+	+	–	High
Thatayatikom et al.	–	–	+/-	+	+	–	+	+	–	High
Tesch et al.	–	+	+/-	+	NA	–	+	+	+	Intermediate
Tessarin et al.	–	–	+/-	+/-	+	–	+	+	–	High
Tillman et al.	–	–	+	+	+	- (children)	+	+	–	Intermediate
Verbsky et al.	+/-	–	+	+	+	–	+	–	+	Intermediate
Vitale et al.	–	–	+	+	+	–	+	+	–	High
Wehr et al.	+	–	+/-	+/-	NA	–	+	+	–	High
Wislez et al.	–	–	+/-	–	+	+ (smoker)	+	+	–	High
Zdziarsky et al.	–	–	+/-	+	+	+ (non smoker)	+	–	–	High

In 27 studies the level of evidence was 4, and in 12 studies the level of evidence of 3. The associated level of practice recommendations was weak in both groups.

## Discussion

To our knowledge, this is the most comprehensive systematic review analyzing treatment efficacy for GLILD in CVID. We show that there is still much uncertainty about the optimal treatment for GLILD and that more basic scientific and clinical research is needed in order to establish the best standard of care.

There are many factors influencing the choice of treatment. Apart from efficacy, risk-to-benefit ratio and patient preference, drug availability and cost may also play a role. Several studies reported that the efficacy of glucocorticoid monotherapy is limited. Other immunosuppressants were often used as second-line therapy with varying results. Rituximab monotherapy and combination chemotherapy with rituximab and azathioprine emerged as promising second-line treatments. Abatacept has been used in patients with CTLA-4 and LRBA mutations, but has not been routinely used in other patient populations as of yet. Finally, HSCT may be an option when other treatments have failed, but reported survival after HSCT in CVID has been poor.

Our findings suggest that glucocorticoids, although widely used as first line therapy, failed to induce remission in 57% (17 individuals) of patients using glucocorticoids (18, 23, 26, 27, 31, 36–38). Treatment with glucocorticoids led to a partial response in 13% (four individuals) and failed to maintain remission in 7% (two individuals) of patients (18, 29). There are, however, also literature reports about the positive effects of glucocorticoids (16, 17, 20, 21). 23% (seven individuals) of all patients using glucocorticoids had resolution of symptoms. It is currently unclear how much reporting bias has occurred in the reports describing the use of for example glucocorticoids for treatment of GLILD. Based on current knowledge, it remains unclear how the benefits of glucocorticoids in some patients may weigh against the side-effects of long-term treatment.

With respect to the category of the (biological) DMARDs, MMF, azathioprine, cyclosporine, sirolimus and infliximab have demonstrated efficacy in single case reports. Yet, because of the anecdotal nature of the studies and the relatively small patient populations they were described in, there is insufficient evidence to make definitive statements. While a previous survey has shown that most physicians agree on the implementation of azathioprine and MMF, there is no consensus as far as other (biological) DMARDs are concerned (9).

We found that rituximab monotherapy was effective in treating GLILD in most cases, although relapses did occur after B cell reconstitution (10, 39). Combination chemotherapy with rituximab and azathioprine is another potential treatment regimen in patients with CVID and GLILD. Our collected data show that this combination of drugs was effective at inducing remission in all cases, even where other therapies had failed (36–38). However, there are also indications that upon prolonged follow-up, relapses may occur (10, 47). The findings on rituximab are in line with published literature which indicates both rituximab and rituximab-based chemotherapy are effective treatments for GLILD in CVID (9). The current literature does not allow to determine whether rituximab monotherapy is superior, equally effective or inferior to rituximab-based combination chemotherapy.

Abatacept is often implemented in the treatment of GLILD in patients with CTLA-4 haploinsufficiency and LRBA deficiency. Results were promising as the drug was effective in most reported cases. Although abatacept is mostly implemented for the treatment of patients with CTLA-4 or LRBA related diseases, it would be interesting to see whether it could be of benefit in other GLILD patient populations as well.

HSCT is a potentially curative treatment for immunodeficiencies and GLILD, yet is associated with the risk of serious complications. Our results show that when successfully carried out, HSCT does indeed lead to resolution of GLILD symptoms in most cases. One exception was two patients in the study by Tesch et al., who developed GLILD after HSCT (59). On the other hand, the reported mortality rate was still relatively high compared to overall survival of patients transplanted for other types of PID. While for patients with CVID and GLILD the survival after HSCT varied between 48% and 70%, in PIDs in general it approaches 90% (61). Furthermore, the procedure of HSCT encompasses immunosuppression as a result of the conditioning and replacement of hematopoietic stem cells, and it is as yet not fully proven which of these two components is responsible for the reduction of GLILD activity after HSCT. There are many factors influencing transplantation outcome, including HLA matching, severity of pre-existing lung disease, infections and the presence of active inflammation in other organs which can make transplant more hazardous. Bone-marrow microenvironment, that is, the complex interplay of local and systemic factors driving and influencing stem cell development, has recently emerged as a potential contributor to the success or failure of HSCT. As pointed out by Troilo and colleagues, approximately half of patients with CVID undergoing HSCT experience incomplete B-cell reconstitution. By studying development and maturation of B-cells of immunodeficient patients with different genetic mutations *in vitro*, the researchers found that patients with a non-supportive bone-marrow niche may not allow for adequate immune cell reconstitution and may have worse outcomes (62). These findings may help in in the prediction of which CVID patients with GLILD could benefit from HSCT.

Furthermore, our study did not find clear differences in treatment responses between children (27 individuals) and adults (228) with GLILD. While mortality is higher in patients with pediatric-onset disease (63) almost all literature reports of children with GLILD showed a positive response to treatment. However, in order to make a clear statement about the prognosis of pediatric-onset GLILD, long-term follow-up data would be required.

### Strengths & Limitations

This is the first review that comprehensively summarizes all peer-reviewed data about the treatment of GLILD in CVID. A systematic approach was implemented according to the internationally recognized PRISMA guidelines that aimed at identifying all existing literature on the treatment of GLILD in CVID. Two databases were searched and, in order to reduce the risk of bias, the screening process was carried out by two independent blinded researchers.

Despite efforts to minimize weaknesses, several limitations need discussion. First of all, there might be bias intrinsic to the published studies. Glucocorticoids are considered first-line treatment for GLILD (9), which could mean that their efficacy is taken for granted and successfully treated patients are under-reported.

Further, the definition of GLILD used throughout this paper may have some limitations. Even though we strictly adhered to the internationally recognized definition of GLILD used by the British Lung Foundation/United Kingdom Primary Immunodeficiency Network, we must acknowledge that GLILD is a spectrum of symptoms and manifestations and that the impact on daily life and response to treatment may differ accordingly. Hence, there is a certain degree of interindividual variation that is difficult to quantify in the absence of detailed and objective information, such as standard radiological scores and pulmonary function tests.

Moreover, we excluded several case reports describing patients with CVID and granulomatous disease, often classified as sarcoidosis, not fulfilling the current GLILD criteria. However, some of these patients may have suffered from GLILD. Indeed, there are several case reports describing patients who were misdiagnosed with sarcoidosis and who were frequently unresponsive to glucocorticoid monotherapy, similarly to the results described in this review (64–66).

Moreover, treatment regimens were strictly defined to enable comparison of the effects of different types of monotherapy. In addition, strict criteria for evaluation of remission of GLILD were formulated. Because of this, small positive effects of treatment might have been underreported in this study.

Finally, long-term effects of medication are seldom mentioned, including the risk of infection linked to the prolonged use of immunosuppressants. This could either mean that the added effect of immunosuppressants in already immunocompromised individuals is negligible or that there is some degree of reporting bias at play. Similarly, little to no side-effects were mentioned in the analyzed literature. However, glucocorticoids are unsuitable long-term therapy candidates because of detrimental effects on metabolism, bone density, growth and behavior. As mentioned previously, the quality of the evidence was relatively low, because none of the included studies had an experimental set-up. The choice of outcome measures was heterogeneous, and often only qualitative assessments were made, thus preventing meta-analysis. Possible confounders were rarely mentioned in the reviewed literature. Hence, it was difficult to make any final recommendations for clinical practice based on the available literature.

### Future Directions

Understanding the cause of GLILD is critical in finding a cure for this disease. About 10-20% of patients with CVID develop GLILD, which suggests that the complication is brought on by a combination of (epi-) genetic and/or environmental factors rather than a single cause (7). It could be postulated that individuals with GLILD are a specific subset of the patient population with CVID, with a susceptibility for lymphoproliferation. Reverse thinking by translating from the bench back to hypothesis formulation can help assemble a workable theoretical framework. If, as is currently thought, GLILD is a form of immune dysregulation, there are potentially two important players, namely T-cells and B-cells (67).

The efficacy of second-line immunosuppressants that selectively target T-cells suggest they have an important role in the pathogenesis of GLILD. On the other hand, the successful use of rituximab in the treatment of the disease supports the idea that B-cells may be important effector cells, either initiating or maintaining inflammation in GLILD. A combined role of T- and B-lymphocytes has also been suggested: superior efficacy of the combination of azathioprine and rituximab compared to rituximab monotherapy would plead in favor of this hypothesis (38).

However, fundamental research into the pathophysiology of GLILD is needed to corroborate any of the above-mentioned hypotheses. In patients in whom monogenetic defects are identified, personalized medicine with individualized treatment strategies could be devised. Histopathological analysis, where available, may support this. Abatacept in CTLA-4 haploinsufficiency and LRBA deficiency is a good example of how personalized medicine is already being implemented in clinical practice.

In order to improve patient care and treatment of GLILD, it is important to screen for the condition, and define the best standard of treatment (9). RCTs are still lacking, because, due to the low incidence of GLILD, it is difficult to recruit sufficient numbers of participants. However, a combined effort by international consortium of medical centers, could allow for standardized data collection on a much larger scale, including pulmonary function tests and a uniform radiographic high-resolution CT scan score. Indeed, studies such as STILPAD are on-going and will inform on this. Until then, uniform standardized reporting on GLILD is crucial. Based on previous literature, this should at least include information on how the GLILD diagnosis was made, dosage and interval of the intervention, treatment-associated side effects (both short- and long-term), pre- and post-treatment CT scores using a universal scoring method, pulmonary function tests including carbon-monoxide diffusion and lymphocyte phenotyping data, ideally using validated tools. Results could provide scientific backup for current treatment strategies and help create new, evidence-based treatment protocols.

## Conclusion

Based on this systematic review of the current literature, which was often of low quality with a high risk of bias, it is impossible to define which therapeutic option is optimal in treating GLILD in CVID.

Corticosteroid monotherapy seems suboptimal for many patients, rituximab monotherapy and combination chemotherapy with rituximab and azathioprine were effective in most reported cases. The use of abatacept has so far been only implemented as therapy for patients with pathogenic CTLA-4 and LRBA mutations. HSCT is the only curative treatment for GLILD, yet not free of risks. While much is left open and uncertain, what has become most evident throughout this review is that there remain many critical knowledge gaps concerning treatment of GLILD. Etiology and optimal treatment for the disease are questions that require urgent answers, as they may lead to better and more specific treatment regimens. In the future, larger well-designed studies evaluating therapeutic strategies should be carried out, with uniform quantitative outcomes.

## Data Availability Statement

The original contributions presented in the study are included in the article/**Supplementary Material**. Further inquiries can be directed to the corresponding author.

## Author Contributions

OL and BS created the search string, selected the articles included in the review, wrote the paper, and created the tables. JM chose the review topic, and guided the research and writing process. JW gave advice about the methodology and reviewed the final text. CC-R and H-eH provided additional raw data which was included in the review. VD, GB, JH, H-eH, HI, HL, ST-L, SP, AR, AS, AV, and KW gave advice during the synthesis of the results, commented on the draft papers, and reviewed the final text. All authors contributed to the article and approved the submitted version.

## Funding

Financial support for this publication was provided by the Louise Vehmeijer Foundation, “e-GLILDnet” and the European Respiratory Society.

## Conflict of Interest

JH and KW co-chair the European Respiratory Society-funded e-GLILDnet Clinical Research Collaboration which is a collaboration with ESID (the European Society for Immunodeficiencies).

The remaining authors declare that the research was conducted in the absence of any commercial or financial relationships that could be construed as a potential conflict of interest.

The reviewer EK declared a past co-authorship with one of the authors CC-R to the handling editor.

## Appendix: Search String

Population: patients with PID and GLILD

Intervention: treatment (pharmacological and/or stem cell transplantation)

Control: no therapy or placebo

Outcome: clinical symptoms, pulmonary function tests, radiologic findings, mortality

### PubMed

“common variable immunodeficiency”[MeSH] OR CVID [Title/Abstract] OR common variable immunodeficiency [Title/Abstract] OR primary immunodeficiency [Title/Abstract] OR GLILD [Title/Abstract] OR antibody deficiency [Title/Abstract] OR granulomatous lymphocytic interstitial lung disease [Title/Abstract] OR granulomatous disease[Title/Abstract] OR interstitial lung disease [Title/Abstract] OR ILD [Title/Abstract] OR granulomatous lung disease [Title/Abstract] OR lymphocytic interstitial pneumonitis [Title/Abstract] OR lymphoid interstitial pneumonitis [Title/Abstract] OR LIP [Title/Abstract]

AND “hematopoietic stem cell transplantation”[MeSH] OR hematopoietic stem cell transplantation[Title/Abstract] OR HSCT[Title/Abstract] OR stem cell transplantation[Title/Abstract] OR SCT[Title/Abstract] OR “abatacept”[MeSH] OR abatacept[Title/Abstract] OR corticosteroid*[Title/Abstract] OR prednisone[Title/Abstract] OR methotrexate[Title/Abstract] OR “mycophenolic acid”[MeSH] OR “mycophenolic acid” [Title/Abstract] OR mycophenolate mofetil[Title/Abstract] OR rituximab[Title/Abstract] OR “azathioprine”[MeSH] OR azathioprine[Title/Abstract] OR immunosuppressant[Title/Abstract] OR immunomodulator[Title/Abstract]

### EMBASE

‘common variable immunodeficiency’/exp OR ‘common variable immodeficiency’:ab,ti,kw OR CVID:ab,ti,kw OR ‘primary immunodeficienc*’:ab,ti,kw OR ‘antibody deficiency’:ab,ti,kw OR GLILD:ab,ti,kw OR ‘granulomatous lymphocytic interstitial lung disease’/exp OR ‘granulomatous lymphocytic interstitial lung disease’:ab,ti,kw OR ILD:ab,ti,kw OR ‘granulomatous lung disease’:ti,ab,kw OR ‘interstitial lung disease’:ab,ti,kw OR ‘lymphocytic interstitial pneumonia’:ti,ab,kw OR ‘lymphocytic interstitial pneumonitis’:ti,ab,kw OR ‘lymphoid interstitial pneumonitis’:ti,ab,kw

AND ‘stem cell transplantation’/exp OR ‘stem cell transplantation’:ti,ab,kw OR ‘hematopoietic stem cell transplantation’:ti,ab,kw OR abatacept/exp OR abatacept:ab,ti,kw OR corticosteroid/exp OR corticosteroid:ab,ti,kw OR prednisone:ab,ti,kw OR ‘mycophenolic acid’/exp OR ‘mycophenolic acid’:ti,ab,kw OR ‘mycophenolate mofetil’/exp OR ‘mycophenolate mofetil’:ti,ab,kw OR methotrexate/exp OR methotrexate:ab,ti,kw OR immunosuppressant:ti,ab,kw OR immunomodulator:ab,ti,kw
